# Heat Induces Oxidative Stress: Reproductive Organ Weights and Serum Metabolite Profile, Testes Structure, and Function Impairment in Male Cavy (*Cavia porcellus*)

**DOI:** 10.3389/fvets.2020.00037

**Published:** 2020-02-26

**Authors:** Ferdinand Ngoula, Fulbert Aime Lontio, Herve Tchoffo, Faustin Pascal Manfo Tsague, Roméo-Marcial Djeunang, Bertin Narcisse Vemo, Frederic Moffo, Nadege Djuissi Motchewo

**Affiliations:** ^1^Animal Physiology and Health Research Unit, Faculty of Agronomy and Agricultural Sciences, University of Dschang, Dschang, Cameroon; ^2^Department of Biochemistry and Molecular Biology, Faculty of Sciences, University of Buea Buea, Cameroon

**Keywords:** heat stress, male guinea pig, oxidative stress, reproduction, heat shock proteins

## Abstract

The present study was designed to evaluate the effects of heat that induces oxidative stress on reproduction organ weight and serum biochemical, testes structure, and function in male guinea pig (*Cavia porcellus*). Forty-eight male guinea pigs with an average weight of 330.56 ± 23.62 g, aged 3–4 months, were distributed into four groups of 12 animals each. One group (control) was maintained to ambient temperature (20–25°C), while other groups (Groups 2–4) were exposed daily for 6 h, to 32 ± 1°C, 39±1°C, and 46 ± 1°C, respectively. All animals were sacrificed after 60 days' exposure and their reproductive characteristics values were determined. Results revealed a significant decrease (*p* < 0.05) of the weight of testes, epididymis, vas deferens, and accessory glands in cavies exposed to the highest temperature investigated (46 ± 1°C), compared to the control animals. There was a significant (*p* < 0.05) reduction of serum testosterone and LH levels in all heat stress-exposed groups (≥46 ± 1°C) when compared to the control group. Heat stress significantly (*p* < 0.05) decreased sperm mobility, sperm count, and testicular antioxidant enzymes, while increasing testicular malondialdehyde content. However, the serum level of HSP-40 increased in the animals exposed to 39 ± 1°C and decreased when the cavies were exposed to 46 ± 1°C. In conclusion, exposure to heat-induced oxidative stress results in impairment of reproduction organ weight and serum biochemical, testes structure, and function in male cavies.

## Introduction

Temperature is one of the most important ecological factors that directly affect the behavior, growth, reproduction, and survival of all animals (1). High temperature is associated to heat stress, which can induce molecular and physiological changes in mammal reproductive organs and therefore affect their reproduction (2). Heat stress affects both female and male reproductive function. It is responsible for embryonic mortality and sperm characteristics impairment (3, 4). For instance, the findings of Yangli et al. (5) reported that chronic heat stress significantly decreased libido, sperm density, semen volume, and microscopic structure of the rabbit testes.

Animal's body responds to heat shock discomfort by overproduction of reactive oxygen species (ROS) (3). Excess ROS induces oxidative stress (6), which disrupts the structure and function of important molecules such as nucleic acids and proteins. Therefore, the maintenance of a dynamic balance between the production and quenching of ROS is critical. ROS production is regulated by the antioxidant defense system comprising enzymes such as superoxide dismutase (SOD), catalase (CAT), glutathione peroxidase, but also non-enzymatic antioxidants such as vitamins E, C, and zinc. Numerous studies have reported that high temperature commonly increases oxidative stress by down-regulating the production of antioxidant enzymes (7, 8) as well as decreased levels of some reproductive hormones (9). Nonetheless, oxidative stress has long been associated to male infertility.

Another mechanism to cope with abiotic stress conditions is the accumulation of key proteins with a protective role. Among them, heat shock proteins (HSPs) are molecular chaperones that have been detected in almost all types of organisms (10). They protect other proteins from denaturation during stress situations (10). HSP synthesis, in general, increases with elevated temperatures. However, above a distinct threshold temperature (typically above 42°C), an inhibition of HSP synthesis occurs, resulting in exponential cell death (11).

Some researchers (2, 3, 5) have previously demonstrated the effects of heat stress on reproductive characteristics in domestic mammals, but information on its effects in non-native species such as guinea pigs remains very scarce. The present study therefore aims to investigate the effects of heat stress on male guinea pig reproductive parameters and its ability to induce oxidative stress.

## Materials and Methods

### Experimental Animals and Design

Forty-eight male cavies (*Cavia porcellus*) weighing 330.56 ± 23.62 g with an average age of 3–4 months were used. The cavies were reared in the Teaching and Research Farm of the University of Dschang (Cameroon), where the study was carried out from April to June 2018. They were identified using numbered earrings and housed in identical cages of dimensions 100 cm × 80 cm × 60 cm (length, width, and height) under 12 h light/day and had free access to feed and water. All experiments were carried out in compliance with the recommendations of the Guide of the National Academy of Sciences on the care and use of laboratory animals (12) and approved by the Department of Animal Science.

The 48 male cavies were divided into four groups (12 animals per group) in terms of body weight, randomly assigned to temperature-controlled cages. One group (control) was maintained to ambient temperature of 20–25°C, while other groups (2, 3, and 4) were exposed to 32 ± 1°C, 39 ± 1°C, and 46 ± 1°C, respectively, for 6 h every day. Animals were subjected to thermal stress for 60 days.

### Data Collection

Twenty-four hours after the last exposure to heat, each animal was anesthetized using ether vapor and blood sample collected by cardiac puncture for determination of hormone levels and HSP-40. Testes, epididymides, vas deferens, and sexual accessory glands were excised out and weighed. The cauda epididymides were weighed and minced in 10 ml of 0.9% NaCl solution (37°C) for sperm characteristics evaluation as follows: 20 μl of the latter solution was placed on a slide and observed (4 files of slide) at 40× magnification under a light microscope, and sperm mobility score was attributed according to the method described by Boiti et al. (13). Sperm count was done using the Thomas hemocytometer. Briefly, defined aliquots of the sperm were dissolved and immobilized in 10% Formol/NaCl solution. After gentle stirring of the sample, spermatozoa were counted microscopically using a hemocytometer chamber in a total of 10 squares out of two chamber fields. Sperm morphological abnormalities (small and big heads, coiled tails) were evaluated microscopically using eosin–nigrosin solution in a total number of 200 spermatozoa (14).

### Histology

The right testis was fixed by immersion in 10% NaCl solution for 1 week and then washed, dehydrated in ascending grade alcohol bath, clarified in xylene immersion, and embedded in paraffin. Sections of 5 μm were stained with hematoxylin–eosin for histological observations under a light microscope (400×).

### Evaluation of Oxidative Stress

For each animal, 15% homogenate of testes (weight/volume) was prepared in 0.9% NaCl solution and centrifuged (3,000 rpm, 30 min), and the supernatant was used to evaluate oxidative stress indicators. Malondialdehyde (MDA) concentration was determined by the thiobarbituric acid method (15), while the superoxide dismustase activity was evaluated according to Dimo et al. (16) method. The catalase (CAT) activity was assessed using the chromic acetate method (17), and glutathione peroxidase (GPx) activity was determined by the potassium iodate method (18).

### Evaluation of Reproductive Hormones, HSP, and Total Cholesterol

Serum levels of testosterone, FSH, and LH were measured using an ELISA kit from Omega Diagnostics (Scotland, United Kingdom). HSP levels in serum samples were determined using guinea pig HSP-40 ELISA kit (ABclonal Biotechnology Co., Ltd, China), as per instructions from the manufacturer. Total cholesterol was determined by colorimetric method using commercial kits CHRONOLAB, Ref. 101-0576.

### Statistical Analysis

Data were analyzed using SPSS IBM statistics software 20.0. Differences between groups were assessed using one-way analysis of variance (ANOVA), and specific differences between pairs of means were assessed by Duncan's test at 5% significance. The mobility and morphology proportion mean and standard deviation of each treatment were determined using descriptive statistics.

## Results

### Effects of Heat Stress on Reproductive Organ Weights in Male Guinea Pig

The weights of the testes, vas deferens, and accessory sex glands (Table 1) significantly declined (*p* < 0.05) in cavies exposed to heat stress when compared to the control group. The weights of epididymis decreased significantly (*p* < 0.05) in animals exposed to the highest temperature (46 ± 1°C) compared to those of the other groups.

**Table 1:** Effects of heat stress on reproductive organ weight in male guinea pig.

**Relative weight of genital organs (g/100 g bw)**	**Levels of temperature (**°**C)**				***p*-value**
	**20–25** ***n* = 12**	**32 ± 1** ***n* = 12**	**39 ± 1** ***n* = 12**	**46 ± 1** ***n* = 12**	
Testis	0.44 ± 0.03^a^	0.34 ± 0.09^b^	0.36 ± 0.04^b^	0.23 ± 0.04^c^	<0.001
Epididymis	0.09 ± 0.01^a^	0.08 ± 0.019^a^	0.08 ± 0.01^a^	0.06 ± 0.01^b^	<0.001
Vas deferens	0.50 ± 0.124^a^	0.24 ± 0.071^b^	0.31 ± 0.08^b^	0.25 ± 0.04^b^	<0.001
Accessory glands	0.05 ± 0.014^a^	0.03 ± 0.01^ab^	0.04 ± 0.01^ab^	0.01 ± 0.01^b^	<0.05

a, b, c *Within the same row, values with the same letters are not significantly (p > 0.05) different. bw: body weight. p: probability; n: number of cavies*.

### Effects of Heat Stress on Sperm Characteristics in Guinea Pig

As shown in Table 2, sperm motility and sperm number decreased significantly (*p* < 0.05) in guinea pigs exposed to high temperatures as compared to the control animals. In addition, the spermatozoa with small and large heads decreased in heat-exposed groups, with respect to the control.

**Table 2:** Effects of heat stress on sperm characteristics in male guinea pig.

**Caudal epididymal sperm characteristics**	**Levels of temperature (°C)**				***p*-value**
	**20–25** ***n* = 12**	**32 ± 1** ***n* = 12**	**39 ± 1** ***n* = 12**	**46 ± 1** ***n* = 12**	
Motility (%)	96.67 ± 0.58	72.50 ± 2.89	65.00 ± 2.16	20.00 ± 0.00	/
Number/cauda epididymis (×10^7^)	6.75 ± 0.25^a^	5.92 ± 0.38^b^	5.58 ± 0.14^b^	0.25 ± 0.00^c^	<0.001
Number/g of epididymal tail (×10^6^)	7.88 ± 0.72^a^	5.44 ± 0.43^b^	4.75 ± 0.50^b^	0.25 ± 0.00^c^	<0.001
Spermatozoa with small and large heads (%)	19.75 ± 0.50	13.00 ± 0.82	16.67 ± 2.89	5.33 ± 0.58	/

a, b, c *Within the same row, values with the same letters are not significantly (p > 0.05) different. p: probability; n: number of guinea pig*.

### Histological Structure of the Testes

**Figure 1:**
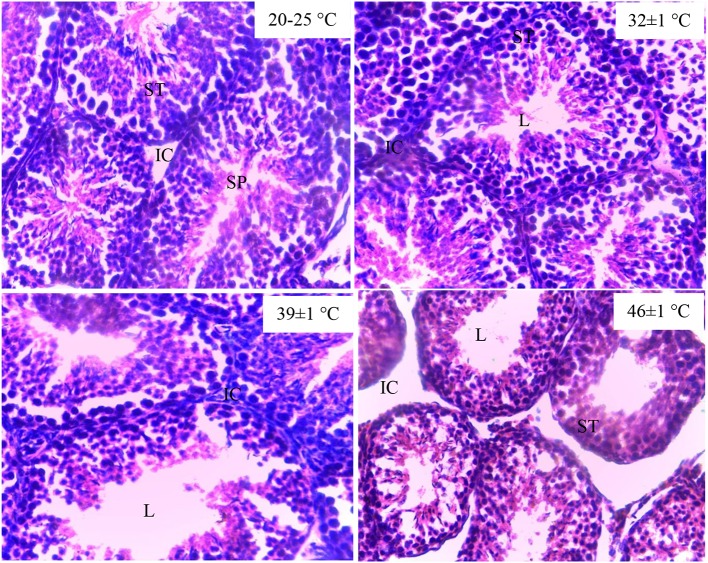
Histological structure of testes in guinea pig exposed to temperature (20–25°C, 32 ± 1°C, 39 ± 1°C). SPZ, spermatozoa; ST, seminiferous tubules; IC, interstitial cells; L, lumen of the seminiferous tubules.

The testes of the guinea pig exposed to 20–25°C and 32 ± 1°C showed normal structure of the seminiferous tubules and interstitial space. The exposure of the cavies to 39 ± 1°C and 46 ± 1°C of temperature destroyed the lumen component of the seminiferous tubules. In addition, the destruction of the interstitial cells was noted in the testes histological structure of the guinea pig exposed to 46 ± 1°C (Figure 1).

### Effects of Heat Stress on Reproductive Hormones and Total Cholesterol in Male Guinea Pig

Serum LH and testosterone concentrations decreased significantly (*p* < 0.05) in animals exposed to heat compared to the control group, while FSH concentration remains unchanged in heat-exposed cavies compared to the control animals. A significant (*p* < 0.05) increase in serum cholesterol was also observed in cavies exposed to 39 ± 1 and 46 ± 1°C when compared to the control group (Table 3).

**Table 3:** Effects of heat stress on serum reproductive hormones and total cholesterol levels in male cavies.

**Hormones and cholesterol concentrations**	**Levels of temperature (°C)**				***p*-value**
	**20–25** ***n* = 12**	**32 ± 1** ***n* = 12**	**39 ± 1** ***n* = 12**	**46 ± 1** ***n* = 12**	
Total cholesterol (mg/dl)	86.87 ± 13.13^b^	83.81 ± 11.76^b^	113.86 ± 6.81^a^	120.73 ± 13.75^a^	<0.001
FSH (mUI ml^−1^)	12.25 ± 2.36	9.01 ± 2.35	10.25 ± 2.22	9.21 ± 1.79	>0.05
LH (mUI ml^−1^)	10.03 ± 2.13^a^	4.88 ± 1.13^b^	6.55 ± 1.21^b^	4.88 ± 1.22^b^	<0.001
Testosterone (ng ml^−1^)	1.41 ± 0.44^a^	0.72 ± 0, 14^b^	0.55 ± 0.14^bc^	0.29 ± 0.07^c^	<0.001

*n: number of guinea pig*.

a, b, c *Within the same row, values with the same letters are not significantly (p > 0.05) different. p: probability. FSH: follicle-stimulating hormone; LH: luteinizing hormone*.

### Effects of Heat Stress on Some Oxidative Stress Indicators in Male Guinea Pig

As shown in Table 4, activities of the antioxidant enzymes, catalase (CAT), superoxide dismutase (SOD), and glutathione peroxidase (GPx), significantly decreased (*p* < 0.05) in animals exposed to heat stress compared to the control group. Conversely, MDA concentration increased with heat stress.

**Table 4:** Effects of heat stress on oxidative stress indicators in male guinea pig.

**Oxidative stress indicators**	**Levels of temperature (°C)**				***p*-value**
	**20-25** ***n* = 12**	**32 ± 1** ***n* = 12**	**39 ± 1** ***n* = 12**	**46 ± 1** ***n* = 12**	
MDA (μM/g of testis)	0.83 ± 0.04^d^	1.14 ± 0.04^b^	1.36 ± 0.01^a^	0.91 ± 0.04^c^	<0.05
GPx (μmol/min/g of testis)	31.68 ± 0.57^a^	28.44 ± 1.31^b^	26.81 ± 0.31^c^	25.03 ± 0.74^d^	<0.001
CAT (μM/min/g of testis)	9.28 ± 0.07^a^	6.29 ± 0.03^b^	5.89 ± 0.05^c^	5.22 ± 0.05^d^	<0.001
SOD (U/min/mg of testicular protein)	1.45 ± 0.07^a^	0.36 ± 0.03^d^	0.75 ± 0.04^b^	0.61 ± 0.05^c^	<0.001

a, b, c, d *Within the same row, values with the same letters are not significantly (p > 0.05) different. p: probability; n: number of guinea pig. MDA: malondialdehyde; CAT: catalase; SOD: superoxide dismutase; GPx: peroxidase glutathione*.

### Effects of Heat Stress on HSP in Male Guinea Pig

**Figure 2:**
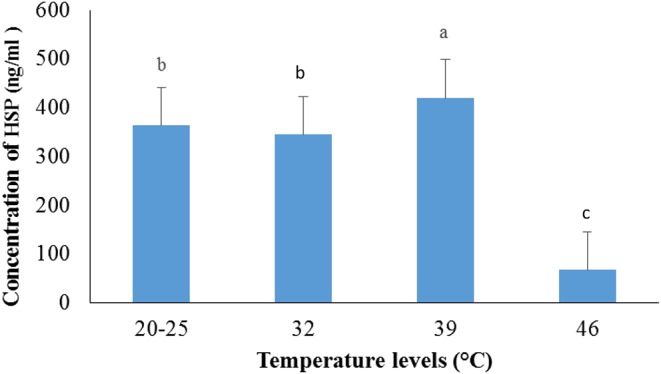
Variation of serum HSP-40 in male guinea pig exposed to different levels of temperature.

The serum level of HSP-40 increased in cavies exposed to 39 ± 1°C temperature (*p* < 0.05) when compared to the control group (20–25°C) (Figure 2), but decreased when the temperature was further increased to 46 ± 1°C (*p* < 0.05).

a, b, c: histogram bars with the same letters are not significantly (*p* > 0.05) different.

## Discussion

In this study, the impact of heat stress on reproductive parameters and its ability to induce oxidative stress in male guinea pigs were assessed. The weights of reproductive organs including testis, epididymis, vas deferens, and accessory sex glands decreased in animals exposed to high temperature (46 ± 1°C) compared to the control. Garrigue (19) also reported decreased reproductive organ weight in mice exposed to high temperature (40 to 42°C) for 60 days. This observation disagreed with the finding of Ngoula et al. (20) who recorded no significant differences in reproductive relative organ weights of younger guinea pigs (aged 2.3 to 3 months) exposed to 45°C. Thus, a temperature increase of 1°C in older male cavies (aged 3 to 4 years) after 60 days of exposure would result in heat stress, which impairs their reproductive organ cells and subsequently reduces their weights. According to Meade et al. (21), aged individuals are more susceptible to heat-adverse effects than young. Furthermore, rodents such as guinea pigs cannot produce sweat to regulate their body temperature under heat conditions (22). They also lack the ability to manufacture their own vitamin C (23). These conditions make them more sensitive to a marginal increase in the tolerable environment temperature. Decrease in testicular weight could be associated to the decrease in the number of Sertoli and germ cells within the seminiferous epithelium (24). Indeed, exposure of the animals to high temperature results into overproduction of ROS, which impairs cell membrane and nucleic acids and subsequently induces an apoptosis. The sperm cells due to their high concentration in polyunsaturated fatty acid are specially predisposed to peroxidation by the ROS. Impairment of testicular function by oxidative stress results in reduction in testicular space of the Leydig cells, which could release steroid hormones and other factors involved in the regulation of the hypothalamus–pituitary–gonadal axis. This could explain the decrease in testosterone, the most important androgen in males mainly produced by testicular Leydig cells under the control of pituitary gland hormone LH. It is worth noting that testosterone is an anabolic hormone that stimulates muscle development in animals and therefore of importance in animal breeding. Snyder et al. (25) observed that testosterone supplementation increases muscle mass and maximal voluntary strength in a variety of clinical and experimental paradigms.

Sperm density and mobility are considered to be important factors affecting fertility (26). Results obtained from this work show a decrease in sperm count and mobility and loss of sperm membrane integrity in guinea pig exposed to high temperatures. These findings are similar to those reported by Yangli et al. (5) in male rabbit exposed to chronic heat stress. Ghasemi et al. (27) and Schwalm et al. (28) observed decreased spermatozoa number in llamas and mice, respectively, following exposure to heat. Other studies also reported adverse effects of heat stress on sperm and viability (3, 4). Heat-induced stress generally led to azoospermia or oligospermia in many animal species (29). Such observation has been justified in the present study by the destruction of the testes lumen components including spermatozoa. The decrease in sperm quality could be a result of the plasma membrane disintegration following the overproduction of ROS induced by heat stress. In fact, spermatozoa are vulnerable to ROS because their plasma membrane contains large amounts of polyunsaturated fatty acids (30, 31). Thus, excessive generation of ROS in semen by abnormal spermatozoa could be a cause of the low sperm quality (31). Besides, testicular size is a good indicator of sperm number (32). Therefore, spermatozoa characteristics obtained are inherent to the reduction in the weight of sex organs. Moreover, reports of Rasooli et al. (33) suggested that exposure to heat stress results in increase of apoptosis in spermatozoa and subsequently reduces testes and epididymis weights.

The present study showed that heat stress caused an increase in testicular MDA levels and the decrease of CAT, SOD, and GPx. MDA, a product resulting from lipid peroxidation, is an important indicator of oxidative stress in cells (34, 35). Thus, its increase implies oxidation of polyunsaturated fatty acid upon exposure to heat stress. The testicular oxidative stress induced by heat could also explain the concomitant decrease in the activities of CAT, SOD, and GPx, which constitute the first line of defense against ROS. The findings of Pahune et al. (36) reported that the latter enzymes protect spermatozoa from oxidative stress. Free radicals produced by heat stress might oxidize the active sites of these enzymes and reduce their activities.

The present study also reveals that the level of HSP-40 in the animal serum after 60 days of heat stress increased significantly in the group exposed at 39 ± 1°C compared to the control group. This is in agreement with previous observations of HSP-60 in the testis of cryptorchid monkeys (37), in rat skeletal muscles after heat stress (38), and in rabbits exposed at 36°C (5). From this study, the level of HSP-40 also decreased significantly at 46°C compared to other treatments. In fact, HSP synthesis, in general, increases with elevated temperatures. However, above a certain distinct threshold temperature (typically above 42°C), an inhibition of HSP synthesis occurs, resulting in exponential cell death (11).

## Conclusion

The results of this study revealed that heat stress caused a drastic impairment on reproductive function in male cavies, illustrated by markedly altered testes structure and sperm characteristics, and decreased the concentration of reproductive hormones and HSP-40 in animals exposed to 46 ± 1°C.

## Data Availability Statement

The datasets generated for this study are available on request to the corresponding author.

## Ethics Statement

Experimental protocols used in this study were approved by the Ethical committee of the Department of Animal Science of the University of Dschang (ECDAS-UDs 20/03/2017/UDs/FASA/DSAES) and was in conformity with the internationally accepted standard ethical guidelines for laboratory animal use and care as described in the European Community guidelines; EEC Directive 86/609/EEC, of the 24th November 1986.

## Author Contributions

FL and FN conceived and designed the research. FN, ND, FL, HT, BV, and R-MD collected the data and carried out data analysis. FN, FL, HT, FPM, FM, and BV wrote the manuscript. FN and FPM reviewed the manuscript. All authors read and approved the final manuscript.

### Conflict of Interest

The authors declare that the research was conducted in the absence of any commercial or financial relationships that could be construed as a potential conflict of interest.
